# Literary Fools.—Guillaume Postel, Christopher Smart, and Others
*"Essai Biographique sur l'Histoire Littéraire des Fous." Par Octave Delepierre. (*Privately printed*.)


**Published:** 1859-04-01

**Authors:** 


THE JOURNAL

OF

PSYCHOLOGICAL MEDICINE

AND

MENTAL PATHOLOGY.

APRIL 1, 1859.

### ART. I.—LITERARY FOOLS.—GUILLAUME POSTEL, CHRISTOPHER SMART, AND OTHERS.*

"INFIRMITY, that decays the wise, doth ever make the better fool," sagely remarks the vanity-stricken Malvolio, in the "Twelfth Night," how truly, we may learn best from the writings of those fools who have been impelled to wield the pen. "I venture to affirm," writes Nodier, "that if a curious book in bibliography has still to be written it is the bibliography of fools, and if a singular, piquant, and instructive library has still to be formed, it is of their works." This observation forms a fitting epigraph to a second essay by M. Delepierre on Literary Fools, than which a happier illustration of the opinions both of the poet and the bibliographer could not well be conceived.

But the bibliographer needs to hasten slowly in the tempting field which a literary history of fools opens out, for fools are of two classes—the positive and the relative. The folly of the former is absolute, and results from the infirmity that decays them; the folly of the latter is relative to the higher degree of wisdom which is supposed to judge. The wise man, however, if he be as prudent as wise, will hesitate before he places his finger upon this or that work, and says this is the result of folly, for it has happened, and might perchance happen again, that the folly of one age has become the wisdom of the next. The bibliographer must therefore take heed that he does not with his pen torture the memory of some unhappy individual in a manner more lasting than that which Campanella underwent at the hands of the Inquisition. "I have been shut up in fifty prisons," writes that philosopher in the preface to his *Atheism Vanquished*, "and submitted seven times to the most severe torture. On the last

* "Essai Biographique sur l'Histoire Littéraire des Fous." Par Octave Delepierre. (*Privately printed.*)

occasion the torture continued forty hours. Bound with tight cords that broke my bones, suspended, my hands tied behind my back, above a sharp piece of wood which devoured the sixteenth part of my flesh and drew away ten pounds of blood, cured by a miracle after six months of sickness, I was thrown into a ditch. Fifteen times have I been placed in judgment. The first time when it was asked: How then does he know what he has never learned? Has he a demon at his command? I replied: In order to learn what I know, I have used more oil than you have drunk wine. At another time I was accused of being the author of the book *The Three Impostors*, which was printed thirty years before my birth. I was again accused of entertaining the opinions of Democritus, I who have written books against Democritus. I was accused of fostering bad sentiments against the Church, I who have written a book on the Christian monarchy, wherein I have shown that no philosopher could have imagined a republic equal to that which was established at Rome under the Apostles. I have been accused of being a heretic, I who have composed a work against the heretics of our times. . . . Finally, I have been accused of rebellion and heresy for having said that there are spots upon the sun, the moon, and the stars, contrary to Aristotle, who makes the world eternal and incorruptible. . . . It was for that they cast me, like Jeremiah, into the dungeon where there was neither air nor light."—(*Cousin :—Cours de l'Histoire de la Philosophie*, t. ii. p. 287.)

Moreover, we must not too hastily add to the list of fools men who have so fretted themselves or been fretted with their zeal for learning and the enemies they had made thereby, that they may, as Pomponatius described himself, be compared to Prometheus bound to Caucasus, devoured by the need of study as by a vulture, unable to eat, drink, or sleep, an object of derision to the foolish, dread to the people, and umbrage to the authorities. "If the wise erred not," says the old proverb, "it would go hard with fools."

M. Delepierre in his present essay confines himself to the class of fools which we have termed positive, and which is formed of individuals who were truly insane. It is not, however, an easy task at all times to draw clearly a line of demarcation between those eccentric and silly authors who crop out in every period, and those authors whose works have been prompted by, or are tinctured with, insanity.

Literary fools may be separated into four divisions—the theological, the literary, properly so called, the philosophical, and the political. Let it not, however, be supposed that the literary records of these fools are invariably fantastical, for not unfrequently they contain fragments expressed, as Polonius would have said, with "a happiness that often madness hits on, which reason and sanity could not so prosperously be delivered of."

The author or authors of *The Spiritual Squirt for Souls Constipated in Devotion,* and *The Spiritual Snuff-Box to make Devout Souls Sneeze,* might well have had a niche in the theological division of literary fools; but these mystical extravagances are out of the direct line of our subject. Coming more strictly within its bounds are the records of one named Paoletti. He was a Jesuit, and was deranged a long time in consequence of his arduous labours as a missionary in South America. He had been in confinement on account of his madness five years, when he composed a work confuting the doctrines of Thomas Aquinas, and he endeavoured to prove that God used the symbolical instruments of the Jewish rites to determine who should or who should not receive the divine favour. He designed a diagram purporting to show the mode in which the holy vessels employed in the Tabernacle were made use of in order to indicate the future lot of the children of Adam relatively to predestination. In an engraving accompanying the work, God is represented surrounded by angels, and presiding at the manipulation of the symbolical vessels: the divine and the human will are represented as two balls moving in a circle, but in different directions, and in the end finishing by meeting in a common centre. Paoletti wrote also another treatise during his madness. In this work he argues that the aborigines of America are the direct descendants of the devil and one of the daughters of Noah; consequently that it is impossible for them to obtain either safety or grace.

More noted than Paoletti is Guillaume Postel, who lived in the sixteenth century. Once a Jesuit, he was dismissed the Order by St. Ignatius on account of his fantastical notions. He was imprisoned many years in Rome; fled to Venice; was accused of heresy before the Inquisition; was declared innocent, but insane; and afterwards made a second journey to Constantinople and Jerusalem. At Rome he was infatuated with an old woman whom some deemed a courtezan, and whom he called his *Grandmother Jane.* He maintained that Jesus Christ had redeemed man only, and that the redemption of woman would have place by the Mother Jane. He endeavoured to prove this opinion in a work written in Italian, and entitled *La Vergine Veneta* (*The Venetian Virgin*); and in another work in French, printed at Paris, and entitled, *The Three Marvellous Victories of the Women of the New World, and how they ought in justice to command everybody, even those who have the Monarchy of the Old World.*

He pretended that the angel Gabriel had revealed to him divers mysteries, and he believed that the soul of St. John the

Baptist had been transfused into him. He asserted also that when he wrote another of his works (*De Nativitate Mediatoris*), he was inspired by the spirit of Jesus Christ, and that he acted only the part of the copyist. He was condemned to be burned alive, by a decree of the Parliament of Toulouse, but he died in 1581 at the Monastery of St. Martin des Champs, leaving behind him many works, some of which are devoted to the fancies which beset him.

About the same epoch lived Geoffroy Vallée, who became a monomaniac when young. He is said to have had as many shirts as there were days in the year, and he was accustomed to send them to be washed in Flanders, at a spring famous for the purity of its waters. In Paris he gave way to dissipation, and when his reason became manifestly altered, his family placed him under guardianship. He then wrote a book, a tissue of nonsense, but for which he was condemned as an atheist, and, with his book, he was burnt at the stake on the 9th February, 1574. But one copy of the book is known to exist, that which formed the basis of the process which led to the author's death. It was made manifest at his examination that he was insane, because he was questioned before a physician.

The title of his work contains several barbarous anagrams, and it scarcely admits of translation. It runs thus—*La Beatitude des Chrétiens, ou le Fléo de la foy, par Geoffroy Vallée, fils de feu Geoffroy Vallée et de Girarde le Berruyer, ausquels noms de père et mère assemblez il s'y treuve : Lere, geru, vrey fléo de la foy bygarrée, et au nom du filz : va fléo règle foy, aultrement guere la fole foy.* "The Beatitude of the Christians, or the Flower of the Faith, by Geoffroy Vallée, son of the late Geoffroy Vallée and of Girarde le Berruyer, which names of father and mother together will be found in it : Bind, take charge of (?) *faith* true flower of the lapsed faith, in the name of the Son : go flower control faith, else cure mad faith."

*Antoine Fusy* or *Fusi,* a Doctor of Divinity of the University of Louvain, takes his place in the category of literary fools, on account of the unintelligible extravagances of his works, one of which is entitled *The Sharp-shooter of the True Church against the Abuses and Enormities of the False.* In a work having the title of *Mastigophorus, or the Precursor of the Zodiac,* he defends a wild medico-physical discovery which he believed that he had made, but which is scarcely fitted for quotation.

*Simon Morin,* an ignorant and illiterate man, was possessed with the errors of the illuminati, and composed several works, one of which, written in 1661, was entitled, *An Evidence of the Second Coming of the Son of Man,* and in it he asserts that he himself was the Messiah. He was condemned to be burned alive, and he suffered at the stake, his works being destroyed with him, on the 14th of March, 1663. The President de Lamoignon, having demanded of Morin if he had written that the new Messiah would pass through the fire, he answered yes, and that it was of him that the prophet had spoken in the fourth verse of the sixteenth Psalm, "*igne me examinasti, et non est inventa in me iniquitas.*" He had promised to rise on the third day, and a multitude assembled at the place of execution to witness the resurrection.

In the theological category we find also *François Dosche,* who tells us at the termination of the title-page of one of his books, that, "not having the means to print it entire, he has, in order to give it to the light, begun with the end, being as anxious to bring forth the truth of God in him as a pregnant woman is to give birth to her infant:" *John Mason* who proclaimed the visible reign of Christ (whose temporal throne was to be established at Water-Stratford, near Buckingham) and who believed that he received a visit from the Lord: and *Jean P. Parizot,* who attempted to demonstrate that in Genesis and the Evangel of St. John it was announced that the three elements of the Trinity were found everywhere in nature. Salt, the generator of all things, represented God the Father; mercury, in its extreme fluidity, God the Son, spread throughout the universe; and sulphur, from its property of uniting salt and mercury, God the Holy Ghost. He was condemned to the stake for the impiousness of one of his works. He deserved the sentence, not for the impiety of the work, but for the excesses which arose out of it.

Other instances might be cited of writers whose brains have been turned by theology anterior to our own time; but, to come nearer to the present day, we may mention *J. A. Soubira,* the self-called *Apostle of Israel, Messiah of the Universe, Poet of Israel, Lion of Jacob,* &c. Among his works are found *The Second Messiah to the Whole World,* (1818. 8vo.); *Counsel to all the Powers of the Earth,* (1822. 8vo.); *The End of the World Predicted by Soubira, its Epoch fixed, that of the Coming of the Messiah of Israel, and of the first day of the Age of Gold, or of the New Terrestrial Paradise,* (8vo.); *The Wandering Jew to his Bankers,* (8vo., 2 pages); "666," (1828. 8vo.), &c. The pamphlet having the sole title of "666," is composed of prose and verse, and the number 666 is placed at the extremity of each line in every stanza. This is the first stanza:—

"Les banquiers de la France . . . . 666

Des organistes de la foi . . . . . . 666

Et des concertes de la cadence . . . 666

Vont accomplir la loi . . . . . . . 666

Et contremenir l'alliance . . . . . 666."

Lastly, a merchant named *Cheneau* of Mennetout-sur-Cher, made himself notorious, in 1848, by several mad works, one of which is entitled, *Instructions how to obtain Children Healthy in Mind and Body, and as perfect as may be.* Before publishing this work, which he designated "the new religious basis and its mode of organization, in which all will recognise the divine power," he had affixed to the walls of Paris a posting-bill, containing a protestation against all oppressors, and headed *The Will of Jehovah in Christ Jesus, sole God, manifested through his servant, Cheneau, merchant.*

After all, was poor Cheneau far wrong in the idea that underlies his system of moral re-organization, that hereditary transmission plays a greater part in morals than is commonly admitted?

If we turn now to instances of literary fools proper, we find, in the seventeenth century, *Nathaniel Lee* writing in one of the cells of Bedlam dramas, and also verses, which latter excited the praise of Addison, but which indicate the madness of the author. It is told of Lee that, while writing one of his dramas, a cloud chanced to overcast the moon, whereupon he cried, "Jove, snuff the moon!"

In the eighteenth century we find *Alexander Cruden,* the author of the well-known *Concordance of the Holy Scriptures.* He was several times confined in lunatic asylums. His insanity, probably induced by disappointed affection, was distinguished by extraordinary attempts to do good in ridiculous ways. After being released from a confinement in the Bethnal-green Asylum, he wrote a whimsical pamphlet, retaliating upon his keepers, and entitled, *The London Citizen exceedingly injured, giving an account of his Adventures during the time of his severe and long Campaign at Bethnal-green, for nine Weeks and six Days, the Citizen being sent thither in March,* 1738, *by Robert Wightman, a notoriously conceited, whimsical man, where he was chained, handcuffed, strait-waistcoated, and imprisoned,* &c. After a subsequent confinement he wrote another singular and wild work, entitled *The Adventures of Alexander the Corrector,*—alluding to his principal employment at the time as a corrector of the press.

About the same period lived *Christopher Smart,* whose insanity did not extinguish a high degree of poetical power which he possessed. He had received a brilliant education at Cambridge, where he took off the prize for the best poem five years in succession. He became insane in 1794, and it was necessary to confine him in an asylum; but although he was deprived of pen, ink, and paper, he composed there a poem of nearly one hundred stanzas to the glory of the Prophet King David. These verses were traced with a key on the wood panels of his chamber. Several of the verses bear the true stamp of the poet, and M. Delepierre thinks that they almost warrant the doubt whether the writer was insane when he composed them. The poem is not included in Smart's collected works, but the following noble stanzas will convey an idea of its character:—

"He sang of God—the mighty source
Of all things—the stupendous force
On which all strength depends;
From whose right arm, beneath whose eyes
All period, power, and enterprise
Commences, reigns, and ends.

"Sweet is the dew that falls betimes,
And drops upon the leafy limes;
Sweet Hermon's fragrant air,
Sweet is the lily's silver bell,
And sweet the wakeful taper's smell
That watch for early prayer.

"Sweeter in all the strains of love,
The language of the turtle dove,
Pair'd to thy swelling chord;
Sweeter, with easy grace endued
The glory of thy gratitude
Respired unto the Lord.

"Strong is the lion—like a coal
His eyeball—like a bastion's mole
His chest against his foes,
Strong the gyre eagle on his sail,
Strong against tide, the enormous whale
Emerges, as he goes.

"But stronger still, in earth and air,
And in the sea, the man of prayer,
And far beneath the tide,
And in the seat to faith assign'd—
Where ask is have, and seek is find,
Where knock is open wide.

"Glorious the sun in mid career;
Glorious the assembled fires appear;
Glorious the comet's train;
Glorious the trumpet and alarm,
Glorious the Almighty's stretch'd-out arm;
Glorious the enraptured main.

"Glorious—more glorious is the crown
Of Him that brought salvation down
By meekness, called thy Son;
Thou that stupendous truth believed,
And now the matchless deed's achieved,
Determined, dared, and done."

Smart died in 1770. He translated the Psalms, Phædrus, and Horace in prose. His poems were published in 1791; Garrick and Johnson favoured him with their friendship, and the last wrote his life.

Had Smart been permitted to have the same liberty as *Edme Billard*, a literary fool who about the same period amused the Parisian public, he might have died as tranquilly. Billard wrote four plays, *The Joyous Moribund* (1779), *Voltaire Appreciated* (no date), *The Weeper in Spite of Himself* (no date), and the *Suborner* (1782). These plays, although evidently written by a person of diseased mind, are not wanting in gaiety.

*Thomas Lloyd* was persuaded that he was the most sublime poet in the world. His *Sketches in Bedlam, or Characteristic Traits of Insanity* (London, 1823), is a work which contains a most extraordinary and heterogeneous melange of malice, pride, talent, lying, vile failings and great qualities. When, during his confinement in Bedlam, he was enabled to obtain paper, he began to write verses; but as it usually happened that they did not please him, he would throw them into his drink to clean them, as he said. Whatever he had in his pockets or that came to hand, his insanity prompted him to mingle with his food: pebbles, tobacco, bits of leather, bones, coals, were thrown into his pottage, after a process which he termed scientific. Whatever he cast in he conceived that it gave some agreeable flavour to the food, and if he had not been watched he would have swallowed everything with the gusto of an Apicius. He announced that his knowledge was universal in tongues, science, history, and music. Although often liberated, it was as often necessary to replace him in confinement. He lived beyond the sixtieth year.

*Johan Carl Wezal*, born in 1747, and who became insane when thirty-nine years of age, wrote several works under the delusion that he was God. Some of these writings were printed under the title *Opera Dei Wezelii W. S. des Gottes.*

A very interesting history is cited in the *Records of Pennsylvania* (Philadelphia, 1802), of a barrister named Milman, whose reason was overturned by the shock experienced from the awfully sudden death of a lady he was about to lead to the altar. She was struck dead by lightning just before the time appointed for the performance of the marriage ceremony. It was necessary to confine Mr. Milman in an asylum; but as he had long periods of tranquillity he was suffered to make excursions into the country. He had lucid intervals, but whilst they continued he could never be left alone three hours consecutively without danger of relapsing into wildness, or becoming fatuous. Previous to his insanity he had manifested no marked imaginative powers, his aptitude being towards the positive and abstract sciences; but during the lucid intervals of his malady he exhibited no inconsiderable degree of fancy, and from time to time he committed to paper certain reflections and descriptions remarkable for vigour and freshness, and the air of pleasantry which runs through them. The following is an example:—

"Nobody has any business to expect satisfaction in a pure country life for two months, unless they have a decided genius for *leisure.* If a man expects to live in a country, of course he must have something to do, and do it all the while. But to gather up yourself and sit down in a plain country-house, without bears and lions about it, without anything to do but to rest; with no marvels or phenomena, but only the good, real, common country; if you mean to be happy in this, I repeat you should have the element of *leisure* very full and powerful within you. You cannot be happy if you are in a hurry. You must not be in a hurry to get up or sit down; you must not be in a hurry to get up in the morning, or to retire at night: you must regard it quite the same thing whether you look at a tree ten minutes or thirty; if you walk out, never must you look at your watch; go till your return; if you sit down upon a breezy fence or wall, it should be a matter of indifference to you whether it be four o'clock, or five, or six. There can be no greater impertinence than to say, 'It is time to go!' There is no such thing as time to a man in a summer vacation.

"Yet amid the tranquil, dreaming, gazing life, one cannot always be quite as serene as one would, For example, this morning, while the dew was yet on the grass, word came that Charley *had got away.* Now Charley is the most important member of the family, and as shrewd a horse as ever need be. Lately he had found out the difference between being harnessed by a boy and a man. Accordingly, on several occasions, as soon as the halter dropped from his head, and before the bridle could take its place, he proceeded to back boldly out of the stable, in spite of the stout boy pulling with all his might at his mane and ears. This particular morning we were to put a passenger friend on board the cars at 8.10; it was now 7.30. Out popped Charley from his stall like a cork from a bottle, and lo! some fifty acres there were in which to exercise his legs and ours, to say nothing of temper and ingenuity. First, the lady, with a measure of oats, attempted to do the thing genteelly. Not he! he had no objection to the oats, none to the hand, until it came near his head, then off he sprang. After one or two trials, we dropped the oats, and went at it in earnest—called all the boys, headed him off this way, ran him out of the growing oats, drove him into the upper lot, and out of it again. We got him into a corner with great pains, and he got himself out of it without the least trouble. He would dash through a line of six or eight boys with as little resistance as if they had been so many mosquitoes! Down he ran to the lower side of the lot, and down we all walked after him—too tired to run. Oh! it was glorious fun! the sun was hot, the cars were coming, and we had two miles to ride to the depôt! He did enjoy it, and we did not. We resorted to expedients—opened wide the great gate of the barn-yard, and essayed to drive him in; and we did it too, almost; for he ran close to it,—and just sailed past, with a laugh as plain on his face as ever horse had! Man is vastly superior to a horse in many respects, but running on a hot summer's day, in a twenty-acre lot, is not one of them! We got him by the brook, and while he drank, oh, how leisurely! we started up and succeeded in just missing our grab at his mane. Now comes another splendid run. His head was up, his eyes flashing, his tail streamed out like a banner, and glancing his head this way and that, right and left, he allowed us to come on to the brush corner, from whence, in a few moments, he allowed us to emerge and come afoot after him down to the barn again. But luck will not hold for ever, even with horses. He dashed down a lane, and we had him. But as soon as he saw the gate closed, and perceived the state of the case, how charmingly he behaved! allowed us to come up and bridle him without a movement of resistance, and affirmed by his whole conduct that it was the merest sport in the world, all this seeming disobedience; and to him I have no doubt it was!"

Two singular examples close M. Delepierre's instances of literary fools proper. In 1834 a M. G. Desjardins published in Paris, under the title of the *First Babylon* (*Première Babylone*), the first part of a vast drama entitled *Semiramis the Great*. The work is composed of five hundred octavo pages, and many passages are printed in Hebrew, Persian, Arabic, and Chaldee, as well as other characters. Some notion of this madly extravagant book may be obtained from the extract which follows, taken from the fifth *Section of Bitterness*, vulgarly called Act, part of which is in verse and part prose. Voices innumerable and cavernous are heard issuing from the profundities of the earth, and the Prince of Prophets, *God's-Judgment*, says to them:—

> Arise! shake from a vast and sluggish wing,
> The eloquent night-dust of three thousand years,—

and from the bottom of the sepulchres in which this vast accumulation of generations lies, comes forth the myriad-voiced answer—

> Behold, in horizontal ranks we raise us!

"Then the kings, princes, and chiefs innumerable of nations commence, helter-skelter, a kind of round or immense chain, supported behind by the stampings and acclamations of the nations. In the ranks are found mingled and carried along both beasts and brutes contemporaries of the ancient actors of this Apocalyptic scene; all creation, every generation of beings brought forth, reptiles, birds, quadrupeds, all flesh multiplying and moving, great lions in the ranks of gigantic warriors, dromedaries, ostriches, giraffes, boas, elevating their long necks, or advancing spirally in the midst of travelling men; lofty elephants, colossal mastodons their eldest brothers, erecting the monstrous serpent of their trunk above the heads and horns of ancient races, princely, royal, and antediluvian. And above all, the stork, the ibis, and great vultures fly, all rolling together the thick waves of their round, all lightened in the travel of their whirling chain, by rays from the red and flaming face of God; and muttering, roaring, and shrieking these words, each in his tongue, whilst revolving :—

"We represent both the storm and the dreadful thunder
Which grumbles around the mount, which corrupts the earth!
During the long horror of a day of chastisement,
We imitate the rigours of the last judgment," &c.

It seems to us, although it does not appear to have struck M. Delepierre's mind, that this scene of Soubira's has been inspired by the Oriental legends respecting the great Solomon, King of the Genii, and the Mahommedan legends of the condition of man in the interval that exists between the resurrection and the judgment. Solomon is described as having at his command the whole of the beasts and birds that have destructive powers, and when he contended with the evil genii on the earth, advanced the beasts of prey,—lions, tigers, leopards, wolves, &c.,—together with elephants, and every other kind of animal capable of taking part in a struggle; in mid-air sailed Solomon sitting upon his magic carpet, and accompanied by myriads of good genii; and above all flew an army of eagles, vultures, and other birds. The Mahommedan legends tell us that at the resurrection of mankind, the genii, and every variety of animal, will be collected on a vast plain (commentators differ as to its locality), and there for a space, some think of forty years, others of fifty thousand, all created beings, rational and irrational, will experience in advance the lot which will be theirs at the last judgment.

The last example of literary fools proper is a *M. Paulin Gagne*, author of several poems, one of which entitled *L'Uniteide, or the Woman-Messiah*, of which the action is placed in the two thousandth year of the Christian era, was published in 1858. It contains a ridiculous agglomeration of fantastic names and absurd verses. Among the *dramatis personæ* are *L'Ane-Archide*, "daughter of Despotism and of Liberty;" *Demounias*, the forerunner of Antichrist; *Panarchie*, the *Dive Insania*, the *Bœuf Apis*, *Archimonde*, and his illustrious spouse *La Presse*, *Patati-culture*, and many other extraordinary personifications, the names of which are even less extraordinary than the verses and ideas accompanying them. For example, turning into anagrams the names of modern socialistic reformers he places them all in presence of *L'Ane-Archide*, who says to them—

Speak!
Wide awake, if I can, I to your dreams will listen.
Speak Pierre Xourd, Nodourp, Urdel Nillor,
Louis Cnalb, George Nas, Narrédisnoc without gold,
Tebac, Ogu without fear, and all ye great apostles,
Who on the head of others aspire to march.

Then the poet expresses, through their mouths, the different systems of these gentlemen in so far as he somewhat loosely comprehends them. The first canto terminates by the entry of the *Woman-Messiah* into Paris.

The second canto contains the same personifications as the first, with the addition of ambassadors from the sun and the moon, inhabitants of the stars, *Auritheocratie*, *Ratiothéie*, &c. The comet *Trouble-tout* (*Trouble-all*) comes also on the scene, and has a discussion with *Ratiothéie*, and sings a song, *le Galop de la Comète*, to the air *Les Défenseurs de la Religion.*

Nations, I come to toll the final hour
Upon the bells of this vast universe!
Already death has hewn out a huge coffin
And made all ready for the mighty convoy:
Tremble, O nations! no longer have ye shelter,
And utter swiftly your most sad adieu!
Tremble, O nations, before my flaming tail!
O nations, wallow in the fierce chaos of fire.

In the third canto *La Socialiforce* has a long discussion with his partisans, which terminates thus:—

I found for aye the golden age of the belly,
Whose pleasant sway our time has much enlarged:
The belly is the fount of revolutions,
And eke creations and destructions.
The Empty-bellies through the long night thunder;
The Well-filled bellies glow with radiant light;
The Hollow-bellies are not worth a jot.
But I will fill them, for they loudly praise me.
Come then, dear friends, and let us hasten swiftly
To trick out feasts that shall astound the world.

The scene of the fifth canto is placed "wheresoever you wish it," and the text is filled with indecent matter. The scene of the thirty-eighth act of the eighth canto is a vast potato-field, and *Potato-culture* opens the scene in a discourse containing seventy-two lines, of which the following is an example:—

Nations and kings, I am Potato-culture,
Daughter of nature and this frying-age;
* * * * * * *
For aye I have adored this dainty fruit,
Once as an extra eaten by the gods.

This tirade ends with:—

In the potato lies the health of all!

In the same scene, *Carotti-culture* also addresses the kings and nations, and sings a parody of the *Marseillaise*, entitled *The Universal Carrot* (*la Carotte universelle*), commencing—

> "Allons, Enfans, de la Carotte
> Le jour de gloire est arrivé.

Chorus:—

> Aux armes, Carottiers, formez vos bataillons,
> Marchons, que la Carotte inonde nos sillons.

M. Gagne tells us in his preface, that "the vast subject of his humanizing and Christian poem should form the universal poesy of humanity, and the school of truth." Madame *Elise Gagne*, the wife of the author, adds an epilogue, in which she tells us that after the reforms indicated in the poem are carried into effect, abundance and happiness will prevail upon the earth.

The poem, indeed, is no pleasantry on the part of its author. "The whole proves," writes M. Delepierre, "that he employed all the resources of his intellect in writing this *chef d'œuvre.*" We must add that the poem is written in rhyme. Other poems written by M. Gagne, are *Le Suicide, La Monopanglotte ou Langue Universelle, Le Délire, L'Océan des Catastrophes.*

In the section of M. Delépierre's Essay devoted to Philosophical and Scientific Fools, Kant is mentioned, who became insane towards the close of his life, and who has a place among literary fools, inasmuch as recently a work has been discovered in Germany, written by the great metaphysician during his insanity. The next illustration is derived from the fifteenth century, towards the close of which lived, at Pisa, one *Gragani,* a physician, who became mad, and during his confinement in a madhouse he wrote a work entitled, *De Philosophiâ Aristotelis,* which was published, to pacify the writer, at Pisa, in 1496. In this curious book, of which one copy alone, in the library of the Vatican, is known to exist, Gragani attempts to prove that the name of Aristotle was a myth, and that that philosopher never had being.

In 1529, a work was published in Florence entitled, *The Anatomy of Language.* It was written by a physician named *Joseph Bernardi* during his confinement in an asylum. Among other curious opinions, he maintained that the whole race of monkeys had the faculty of speech, but that they carefully kept the gift secret. He drew upon the walls of his cell an anatomical diagram of a monkey's throat, and sought to prove that the construction clearly showed the faculty of speech and even of song. Bernardi asserted that in the first editions of the voyages of Marco Polo, it had been well established that monkeys could sing. What added to the curiosity of all this was, that a Jesuit, Father

Cremoni, wrote a refutation of Bernardi's treatise, and maintained that, although the work of his adversary was well written, the thesis was contrary to the testimony of Holy Writ, and consequently could not be true. How aptly does the biting remark of Jaques apply here—

> "The wise man's folly is anatomised,
> Even by the squandering glances of a fool."

Bernardi lived ten years after the publication of his work, but he never fully recovered his reason.

In 1622, there appeared at Salamanca, under the title of *De Philosophia*, a work written by *Miguel de Flores*, formerly a professor in the university of that city. He had become insane in consequence of concussion of the brain, occasioned by a fall from a carriage. The insanity continued many years, but as he was peaceable, he was suffered to be at liberty. His mania was characterized by an incessant desire to write; and he would carry his manuscripts along with him, stopping the passengers in the streets to read to them his lucubrations. Four years before his death, his friends published one of his essays, and "it is remarkable," writes M. Delepierre, "since that in it is contained the germ of the system developed in our days, under the name of the *atomic theory*, by Father Boscovitch, Dr. Priestley, and others. De Flores represents the Deity as occupying the centre of creation, and all things created as concentric circles, more or less removed the one from the other. Eccentric engravings give an idea of the theory of the author. They depict the Divinity setting in motion all things by the mechanical action of the arms and legs."

*Robert Hall* finds a place in this section, and also *Thomas Wirgman*, who was the author of many works, and who dissipated a great fortune in printing them.

*Wirgman* lived not long ago, and among other freaks, he addressed a letter to George IV., in which he declared that if the principles set forth in his book, the *Devarication of the New Testament*, were not adopted, neither the king nor his subjects would be saved in the other world. The title of the work named runs thus:—"*The development of celestial power, the aggregate of spiritual existence, the sublimity of creative energy, the positive realization of voluntary action, and the blended harmony of supreme wisdom, truth, and goodness.*"

Wirgman's mental failing was not manifested in his writings only, but also in the mode in which his books were fabricated. He had paper made in such a fashion that each leaf of his books was of divers colours; and if the colours did not happen to please him, he would have other paper prepared. He would also frequently change the arrangement of a book in passing through the press. Thus it happened that the book just named, although possessing only 400 pages, cost him £2276 sterling.

Another of Wirgman's works was entitled, *The Grammar of the Five Senses,* and purposed to be a course of metaphysics for infants. The work is illustrated by nineteen coloured diagrams, and of it the author states: "When this (grammar) is adopted, virtue will supersede crime, and establish peace and harmony on earth."

Wirgman was a goldsmith by trade, and he had retired from business with a fortune of £50,000. This was altogether wasted in printing his books, and he died destitute. (*Essay on Bluet d'Arberes,* by M. Delepierre, p. 11.)

Last in the list of philosophical fools is *William Martin,* a brother of *Jonathan Martin,* who set fire to York Cathedral. His first work was entitled, *A New System of Natural Philosophy on the principle of Perpetual Motion*—Newcastle, Preston, 1821. In the title-page he designates himself *Natural Philosopher;* and in the preface he tells us, that having in vain attempted to solve the problem of perpetual motion mechanically, he renounced the subject as impracticable. But the very evening of the day on which he had come to this decision, he had a dream, partly strange and terrible, partly very agreeable; and from this dream he awoke perfectly convinced that God had chosen him to discover the great secondary cause of all things, and the true perpetual motion. Martin wrote several books.

From M. Delepierre's section on Political Fools, we shall quote only three examples.

One *Demons* in the sixteenth century distinguished himself by two works, the title of one of which is as follows: "*The disputative and potential Sextessence obtained by a new method of distillation, according to the precepts of white magic and invocation of Demons, counsellor of the presidial* [an inferior court of judicature] *of Amiens, as well to cure the hæmorrhage, wounds, and venereal ulcers of France, as to change and convert things noxious and abominable into things good and useful.*" (Paris, 1595, 8vo.).

*Francois Davenne,* who believed among other things that he ought to supplant Louis XIV., and who wrote several very curious tracts, proposed two modes in which to demonstrate his sovereign puissance and royal authority. "Take," said he, "the Cardinal, the Regent, the Duke of Orleans, the Princes, the coadjutor, and those whom the world esteems most holy; light a furnace, throw all into it, and let the individual who comes out without injury from the flame, like a phœnix renewed, be considered the protégé of God, and be ordained prince of the people."

The second proposition, made lest the former one should not be accepted, runs thus:—"Let the Parliament decree my death for having dared to tell the princes truth. Let them execute me, and if God saves me not from their hands in a supernatural manner, let my memory be blotted out. If God does not deliver me from the hands of the executioners, there will be an end of the matter; but if the supernatural arm snatches me from their claws, let them be sacrificed in my stead."

In his *Plea of the Eternal Wisdom,* he has the following quaintly expressed thought: "My soul, I immolate thee upon the scaffold of my ideas, with the hand of my desires, by the sword of my resignation."

In 1848, one *Herpain* of Genappe, whose mind had been deranged by ideas of social progress, under the pseudonym of Usaner, published a little work in 18mo., developing a theory of language which he termed *Langage physiologique.* He sent copies of the work to all the legislative assemblies of Europe. That destined for the English Parliament, was addressed *To the Legislators of the great English Nation by their servant Herpain, author.*" In a note at the end of the introduction he suggests the use of certain ciphers in place of the letters ordinarily made use of; for example, "Stat5ng facto opro lit2al ni, ni foʌ2al ovo otano," &c. Fortunately he adds a translation, which is not a bad example of his style of writing:—"Immediately that your majestic presence had lit up the nothing (néant), the nothing became the medium of existence. Then you willed to sway favourably the essences, and the principles of beings were produced by your generous fecundity," &c.

Thus far we have dealt with the literature prompted by insanity, and which is a manifestation of the loss of mental equilibrium. But there is another class of literature of the insane. In several of the lunatic asylums in this kingdom literary composition is encouraged as a curative occupation for the inmates. The Crichton Royal Asylum, in Scotland, has its journal, *The New Moon,* edited, conducted, written, and printed by the lunatics in the establishment. This journal is issued monthly, and has been in existence many years. Some years ago a series of *Memoirs of Mad Poets, Mad Philosophers, Mad Kings, Mad Churls, by the inmates of the Crichton Institution,* were published, and more recently a small volume of poems by the lady patients was printed. The Royal Edinburgh Asylum for the Insane has also its monthly journal, *The Morningside Mirror,* which has been regularly published about twelve years. This journal is also entirely written and printed by the patients. In the Hanwell Asylum literary composition is also encouraged.

Two poetical extracts from *The New Moon,* illustrative of that happiness of expression which often madness hits on, may fittingly close our illustrations of the literature of madness :—

I.

*On the Death of my Bulfinch.*

Oh, couldst thou know, my little pet,
How much thine absence I regret!
Ah! 'twas a day like this
When thou into my little room
To cheer me with thy voice didst come,
Which now I hourly miss;
And 'neath this shade of woe, alone,
Lament my little Goldie gone.

Whene'er thou saw'st me shut within
My room, thou cheerily wouldst sing,
And all thy art employ;
At thy lov'd voice, so sweet and clear,
All care would quickly disappear—
My sadness turn to joy;
And all the trouble of my lot
Be dissipated and forgot.

Wise people do, I know, believe
That birds, when they have ceased to breathe,
Will never more revive;
But—though I cannot tell you why—
I hope, though Goldie chanced to die,
To see him yet alive!
May there not be—if Heaven please—
In Paradise, both birds and trees?

I've had such dreams—they may be true:
Meantime, my little pet, Adieu!

II.

Go! sleep my heart in peace!
Bid fear and sorrow cease:
He who of worlds takes care,
Our heart in mind doth bear.

Go! sleep my heart in peace!
If death should thee release,
And this night hence thee take,
Thou yonder wilt awake.

This last poem might have been written by Herrick.

It may be due to M. Delepierre to express our high appreciation of his admirably written and daintily printed essay, but it is hardly just to our readers, seeing that the great majority of them can only know it through the means of our imperfect abstract, and consequently we might unhappily excite a desire which could not be gratified. The essay is printed for private circulation, and the last line on the last page runs thus:—" *Nota.—Cet Essai n'a été tiré à part qu'à* 50 *exemplaires.*"

---

